# Association of Group B Meningococcal Vaccine Receipt With Reduced Gonorrhea Incidence Among University Students

**DOI:** 10.1001/jamanetworkopen.2023.31742

**Published:** 2023-08-31

**Authors:** Steve G. Robison, Richard F. Leman

**Affiliations:** 1 Oregon Public Health Division, Portland, Oregon

## Abstract

This case-control study examines reported cases of gonorrhea among recipients of meningococcal group B vaccine at 2 universities in Oregon.

## Introduction

In 2017, New Zealand researchers reported that a meningococcal group B vaccine (MBV) appeared to reduce gonorrhea incidence, with vaccine effectiveness of 31%.^1^ These researchers compared population-level gonorrhea rates with rates of chlamydia, an unrelated sexually transmitted infection. *Neisseria meningitidis* and *Neisseria gonorrhoeae* are closely related genetically, and the MBV was found to target outer membrane vesicles (OMVs) common to both.^1^ This finding was initially controversial as, previously, no effective vaccine for gonorrhea had been found. However, following broader use of OMV-based MBVs, several retrospective, population-based studies successfully replicated the New Zealand finding using chlamydia or other vaccines as controls.^2,3,4^ Currently, 2 MBVs are available in the US: MenB-4C (OMV-based) and MenB-FHbp (not OMV-based). Mass vaccination campaigns prompted by group B meningococcal outbreaks at University of Oregon in 2015 and Oregon State University in 2016 each used both available MBVs. Vaccination was confined to students, staff, and some medical and public health personnel. We assessed whether receipt of OMV-based MBV was associated with subsequent lower gonorrhea prevalence than receipt of non–OMV-based MBV.

## Methods

The Public Health Institutional Review Board covering Oregon’s Public Health Division approved this case-control study’s use of public health data. We followed the STROBE reporting guideline.

Study populations were vaccine recipients aged 18 to 29 years who were reported to Oregon’s ALERT Immunization Information System by on-campus or community-based practitioners near the affected universities. Gonorrhea cases were determined by linking immunization to disease reports based on names, birthdates, and other demographic characteristics, from 1 month to 2 years after vaccination or study end (March 31, 2018). Gonorrhea case reporting is mandatory in Oregon. The association of vaccination with changes in gonorrhea prevalence was estimated among recipients of each MBV type. Because older students might be lost to later disease tracking due to graduation and departure from Oregon, we examined vaccine effectiveness separately for students aged 18 to 19 years.

Study data were analyzed in January 2023 using Stata v17 (StataCorp LLC) and open-source WINPEPI v11.65, with 95% CIs reflecting 2-sided tests. As differences between university populations were a potential confounder, a university variable was tested for significance through logistic regression.

## Results

Study populations included 15 760 recipients of 1 or more OMV-based MBV (8510 females [54%], 7250 males [46%]; median [IQR] age at receipt, 19.3 [18-20] years) and 15 212 recipients of 1 or more non–OMV-based MBV (8519 females [56%], 6693 males [44%]; median [IQR] age at receipt, 19.4 [18-20] years). Overall, 53% of non–OMV-based MBV recipients received more than 1 dose vs 57% of OMV-based MBV recipients. Twenty-four cases of gonorrhea were reported in OMV-based MBV recipients vs 44 cases in non–OMV-based MBV recipients. The OMV-based MBV was 47% (95% CI, 13%-68%) effective in preventing gonorrhea among recipients aged 18 to 29 years.

Among those aged 18 to 19 years, 12 gonorrhea cases were reported in OMV-based MBV recipients and 27 in non–OMV-based MBV recipients (vaccine effectiveness, 59%; 95% CI, 20%-79%). Vaccine effectiveness for recipients of 2 or more vaccines vs 1 vaccine was 11% (95% CI, –0.98% to 0.60%).

## Discussion

Results were consistent with other study findings that OMV-based vaccines may offer protection against gonorrhea.^1,2,3,4^ Because both MBVs were used at each university, this study was able to reduce selection bias inherent in retrospective, population-based studies. Study limitations included low numbers of gonorrhea cases, likelihood of students graduating and leaving the disease-reporting area, and shifts in gonorrhea risk over time. Baseline gonorrhea rates for the 2 universities could not be assessed, and differences in rates could introduce bias. Given the global burden of gonorrhea, further investigation of OMV-based vaccination technology may be warranted.^5,6^

## Notes

Supplement.
Data Sharing Statement
