# Supplementary material for: Association of Group B Meningococcal Vaccine Receipt With Reduced Gonorrhea Incidence Among University Students
Source: JAMA Netw Open. 2023 Aug 31;6(8):e2331742. doi: 10.1001/jamanetworkopen.2023.31742 (PMC10472183; doi:10.1001/jamanetworkopen.2023.31742)
Supplement: Supplement. — Data Sharing Statement [file jamanetwopen-e2331742-s001.pdf]

Data Sharing Statement

Robison. Association of Group B Meningococcal Vaccine Receipt With Reduced Gonorrhea Incidence Among University Students. *JAMA Netw Open*. Published August 31, 2023. doi:10.1001/jamanetworkopen.2023.31742

Data
Data available: No

Additional Information
Explanation for why data not available: Individual-level data for Oregon gonorrhea cases cannot be publicly released per Oregon Health Division standards and IRB guidance.
